# Assessing Tumor Oxygenation for Predicting Outcome in Radiation Oncology: A Review of Studies Correlating Tumor Hypoxic Status and Outcome in the Preclinical and Clinical Settings

**DOI:** 10.3389/fonc.2017.00010

**Published:** 2017-01-25

**Authors:** Florence Colliez, Bernard Gallez, Bénédicte F. Jordan

**Affiliations:** ^1^Biomedical Magnetic Resonance Group, Louvain Drug Research Institute, Université Catholique de Louvain, Brussels, Belgium

**Keywords:** tumor oxygenation, oximetry, tumor hypoxia, hypoxia imaging, radiotherapy outcome

## Abstract

Tumor hypoxia is recognized as a limiting factor for the efficacy of radiotherapy, because it enhances tumor radioresistance. It is strongly suggested that assessing tumor oxygenation could help to predict the outcome of cancer patients undergoing radiation therapy. Strategies have also been developed to alleviate tumor hypoxia in order to radiosensitize tumors. In addition, oxygen mapping is critically needed for intensity modulated radiation therapy (IMRT), in which the most hypoxic regions require higher radiation doses and the most oxygenated regions require lower radiation doses. However, the assessment of tumor oxygenation is not yet included in day-to-day clinical practice. This is due to the lack of a method for the quantitative and non-invasive mapping of tumor oxygenation. To fully integrate tumor hypoxia parameters into effective improvements of the individually tailored radiation therapy protocols in cancer patients, methods allowing non-invasively repeated, safe, and robust mapping of changes in tissue oxygenation are required. In this review, non-invasive methods dedicated to assessing tumor oxygenation with the ultimate goal of predicting outcome in radiation oncology are presented, including positron emission tomography used with nitroimidazole tracers, magnetic resonance methods using endogenous contrasts (*R*_1_ and $R_{2}^{*}$-based methods), and electron paramagnetic resonance oximetry; the goal is to highlight results of studies establishing correlations between tumor hypoxic status and patients’ outcome in the preclinical and clinical settings.

## Introduction

**Figure 1:**
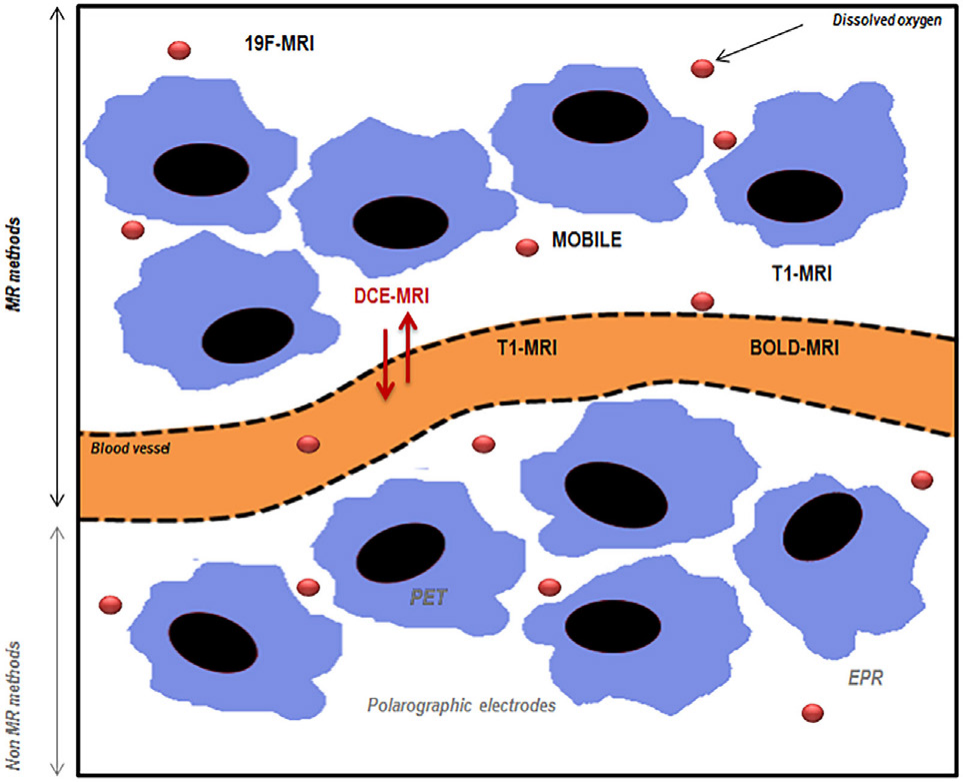
**Schematic representation of magnetic resonance (MR) and non-MR methods used to assess tumor oxygenation**. PET, positron emission tomography; EPR, electron paramagnetic resonance; MRI, magnetic resonance imaging; DCE-MRI, dynamic contrast-enhanced magnetic resonance imaging; BOLD-MRI, blood oxygen level-dependent imaging; MOBILE, mapping of oxygen by imaging lipids relaxation enhancement. Adapted from Price et al. (11).

The effects of chemotherapy and radiotherapy have long been known to be affected by hypoxia (1, 2). Irradiation of normoxic tissues induces water ionization and the formation of radicals such as reactive oxygen species which are able to react with DNA and form DNA radicals. In the absence of oxygen, these radicals can easily be stabilized by cell “scavengers” in order to protect DNA. However, when oxygen is present, the DNA radicals react with oxygen and the damage is fixed. This reinforcement of the X-rays’ efficiency in the presence of oxygen is known as the “oxygen enhancing effect” (3). The “oxygen enhancement ratio” is the ratio of doses required to obtain the same cell survival under hypoxic and aerobic conditions. This value for mammalian cells varies from 2.5 to 3.0 (1, 4), indicating that hypoxic tumor cells will require a dose 2.5–3 times higher to be killed than normoxic cells. Radioresistance is considered maximal at 0.2 mmHg (corresponding to anoxia) and decreases progressively to 20 mmHg, which is the oxygen concentration at which hypoxia-induced resistance is almost nil (4). There are therefore two possible strategies for improving the curative effect of radiotherapy on hypoxic cells: alleviating hypoxia by increasing oxygen availability and increasing the dose of irradiation on hypoxic tumors. From a meta-analysis gathering 10,108 patients with solid tumors and observation of clinical practice, Overgaard concluded that “*Ample data exist to support a high level of evidence for the benefit of hypoxic modification. However, hypoxic modification still has no impact on general clinical practice*” (5). The unavailability of biomarkers as well as the lack of an ideal method for assessing tumor hypoxia, and for monitoring tumor response to radiosensitizers alleviating hypoxia, are issues that prevent the selection of patients who could benefit from increasing the pO_2_ level. The ideal method for patient stratification should be non-invasive, available in both preclinical and clinical settings, repeatable over a short period of time in order to monitor both chronic and acute hypoxia before and during the course of radiotherapy, quantitative from 0 to at least 40 mmHg, widely available in imaging centers, and predictive of the radiotherapy outcome. Finally, this method should provide a parametric value which is easily convertible into a dose of irradiation. Up to now, despite the efforts of scientists, no technique has met all these criteria. Indirect exogenous and endogenous markers for immunohistochemical detection of tumor hypoxia as biomarkers for personalized radiation oncology have recently been reviewed (6), following a previous large-scale review of hypoxia imaging methods in 2012 (7). Reviews with a special focus on preclinical assessment or the imaging of hypoxia have also provided a full description and technical details regarding each methodology (8, 9). Finally, a recent review addresses functional MRI (fMRI) methods in the field of radiation therapy of head and neck tumors (10). This article reviews the results of preclinical and clinical studies acquired using non-invasive imaging methods to assess tumor oxygenation in an attempt to establish correlations with patients’ outcome (according to the oxygen level in their tumors), with special emphasis on preclinical *quantitative* methods, such as electron paramagnetic resonance (EPR) oximetry and clinically translatable endogenous contrast magnetic resonance (MR)-based methods, which have so far been less validated than positron emission tomography (PET)-based methods (Table 1). Cross-validation studies between methods and with quantitative methods are also presented in order to better establish the relevance of each oximetric method. A first section is dedicated to polarographic electrodes that have pioneered *in vivo* oxygen measurements and provided the first human demonstration of the occurrence of hypoxia in human tumors. This article summarizes and assesses the value of MR and non-MR methods used to assess tumor oxygenation in order to predict the outcome of radiation therapy (Figure 1).

**Table 1:** **Oxymetric studies linking hypoxia and radiation therapy outcome**.

Oxymetric technique	Animal studies	Reference	Clinical studies	Reference	Cross-validation with *quantitative* oxymetric methods?	Reference
Eppendorf electrodes	C3H mammary tumors: significant difference in local tumor control between the fraction of hypoxic values (<2.5 mmHg) and less hypoxic tumors	(36)	Prostate cancer study (*n* = 57): 8-year survival is 78% for moderately hypoxic tumors and 46% for severe hypoxic tumors	(16)	n.a.	

			Head and neck cancer study (*n* = 35): 2-year locoregional control is two times lower for hypoxic tumors (i.e., with 15% of readings <2.5 mmHg)	(15)		

PET ^18^F-MISO	FaDu hSCC xenografts: prognostic value of pretreatment ^18^F-MISO hypoxic volume; SUVmax was not associated with local control	(25)	5 head and neck studies (*n* = 45; 73; 12; 17; 15)	(21, 24, 26, 27, 29)	Mixed results	(23)
			4 studies reported correlation between ^18^F-MISO hypoxia and outcome 1 study reported a lack of correlation		Lack of correlation with Eppendorf measurements in head and neck tumors	

PET ^18^F-FAZA	Rhabdomyosarcoma: lower uptake linked to better local tumor control at 90 days post-irradiation	(36)	Head and neck cancer study: DAHANCA trial (*n* = 40), high tumor uptake is correlated to lower disease-free survival	(38)	Positive results	(38)
					Validated with EPR oximetry in the preclinical setting (rat rhabdomyosarcomas)	

	9L glioma and rhabdomyosarcoma: significant correlation between ^18^F-FAZA T/B and tumor growth delay	(37)				

PET ^18^F-FETNIM			1 head and neck cancer study (*n* = 21)	(21)	NO (but compared with other nitroimidazoles)	(44, 45)
			2 lung cancer studies (*n* = 26; 32)		Comparison with F-MISO: positive response under hyperoxic breathing challenge in C3H murine tumors Comparison with FAZA: positive correlation in murine mammary tumors	
			1 cervical cancer study (*n* = 16)			
			1 esophageal cancer study (*n* = 28)			
			High fractional hypoxic volumes, uptake, or baseline SUVmax correlated with PFS, OS, or clinical response			

PET ^60^CU-ATSM	Canine sinonasal tumors: lack of correlation between Cu-ATSM uptake and outcome	(51)	3 cervical cancer studies (*n* = 14; 15; 38)	(21, 48–50)	Mixed results	(40, 57–63)
			2 head and neck cancer studies (*n* = 15; 11)		Comparison with F-MISO, EF5, or pimonidazole: no link with hypoxia in different tumor models or in response to hyperoxic challenges Comparison with Eppendorf electrodes: correlation with hypoxia in FaDu tumors but not in HT29 tumors	
			3 lung cancer studies (*n* = 19; 22; 7)		Potential link with tumor redox status	
			1 rectal cancer study (*n* = 19)			
			Tumor uptake is inversely related to PFS or disease specific free survival Hypoxic tumor volume and hypoxic burden (=HTV × SUVmean) related to PFS			

Dynamic contrast-enhanced magnetic resonance imaging	Melanoma xenografts: low *k*^trans^ is correlated with increased radioresistance	(78)	Cervical cancer study: *k*^trans^ and *A*_Brix_ parameters correlated with poor outcome	(80)	Mixed results	(82)
					Comparison with Eppendorf electrodes: correlation between max DCE signal enhancement and median pO_2_ in cervical cancer patients	

	Cervical cancer xenografts: basal *k*^trans^ correlated to the outcome of RT; skewness (heterogeneity) in *k*^trans^ distribution correlated with poorer outcome	(79)			Comparison with pimonidazole Correlation between “poor perfusion” parameters and hypoxia (pimonidazole staining) in head and neck cancer patients Lack of correlation in glioma mice xenografts and glioma patients	(83, 84)

	Mouse fibrosarcoma: none of the tested DCE parameters (*k*^trans^, *v*_p_, *K*_ep_, % of perfused voxels) were related to RT outcome	(69)				

$R_{2}^{*}-T_{2}^{*}$	G3H prolactinomas (rats)	(100)	Cervical cancer study: basal $R_{2}^{*}$ was predictive for RT response	90	Mixed results	(96–98)
	RIF-1 fibrosarcomas (mice)				Lack of quantitative relationship between fluorescence quenching fiber optic probes pO_2_ values and $\Delta T_{2}^{*}$ values Correlation between pimonidazole and high $R_{2}^{*}$ in prostate cancer Inverse correlation between pimonidazole and $R_{2}^{*}$ in mammary tumors	
	$\Delta R_{2}^{*}$ was predictive for a transient reduction in tumor size; low baseline $R_{2}^{*}$ was linked to a small reduction in tumor size					

*R*_1_–*T*_1_ of water protons	Dunning R3327-AT1 rat prostate	(92)			Mixed results	
	A large increase in *R*_1_ response to hyperoxic challenge was linked to a longer tumor growth delay after radiation therapy				No study addressing potential correlations between *R*_1_–*T*_1_ and quantitative pO_2_ measurements	

*R*_1_–*T*_1_ of lipid protons	9L glioma	(101)			Mixed results	(101, 123)
	Water and lipids *T*_1_ are less predictive of RT outcome than $R_{2}^{*}$ in this model				Comparison with EPR oximetry Positive correlation in mammary tumors Lack of correlation in rat rhabdomyosarcoma and 9L glioma	

Combined *R*_1_ and $R_{2}^{*}$ MRI	Dunning rat prostate tumors	(121)				
	Useful factors to predict tumor response to hypofractionation					

EPR oximetry	C6 and 9L glioma	(151)				
	pO_2_ assessed after a first course of RT was a prognostic indicator of differential response to RT between the two glioma models					

	TLT and FSaII syngeneic tumors	(69, 76, 130, 132, 136–146, 148)				
	pO_2_ assessed during/after administration of treatments able to alleviate tumor oxygenation was predictive of the outcome of RT when administered during this window of reoxygenation					

## Polarographic Oxygen Electrodes

Polarographic electrodes are probes that can be introduced directly into the tissue of interest. The reduction of oxygen at the cathode extremity will generate a detectable current proportional to the pO_2_. The electrodes’ measurements provide histograms of pO_2_, describing the frequency of pO_2_ measurements registered during a defined period of time and corresponding to the mean oxygen level for 50–100 cells that are located around the polarographic electrode (12). Since this technique requires the insertion of the probe inside the tumor, the tissue itself is damaged and a delay is necessary before measurement to allow for stabilization. This also prevents repeated-measurements experiments on the same site and limits the application of the electrodes to accessible tumors. Moreover, as the operation of the polarographic electrodes requires oxygen, the signal-to-noise ratio will obviously decrease with the oxygen concentration, making measurements difficult under severe hypoxia. Since they are invasive and since their function is oxygen consuming, they cannot be chosen as the ideal method for tumor oxygenation measures. The Eppendorf electrode system (which was commercially available) has been developed to limit this consumption effect: the electrode is moved through the tissue of interest and an oxygen measurement is registered every 0.4 mm (after a 0.7-mm step forward and a 0.3-mm step backward). Reducing the delay after the back-step to a minimum helps to ensure negligible consumption of oxygen by the electrode and to decrease the tissue compression artifact (13). The polarographic electrodes have the advantage of providing real-time measurements that can be static or moving (in the case of Eppendorf electrodes). Despite their limitations, the polarographic electrodes have been widely used as a “gold standard” in preclinical and clinical experiments. With regard to clinical use, data from more than 125 clinical studies are available (14). It was shown in 2005 in a head and neck study involving 397 patients that tumor hypoxia assessed using Eppendorf electrodes was associated with a poor prognosis (15). Eppendorf electrodes have also highlighted that the outcome of patients with prostate cancer is linked to the level of tumor hypoxia. The 8-year survival was found to be 78% for patients with moderate hypoxia but just 46% for patients with severe hypoxic tumors; these results were independent of well-established risk factors such as tumor stage, Gleason score (defining the prostate tumor grades), prostate-specific antigen, perineural invasion, serum hemoglobin level, and hormonal therapy use (16). The prognostic value of tumor pO_2_ Eppendorf measurements was less clear in a multicenter human cervix carcinoma study involving 127 patients (17). Finally, as a “gold standard” method, the polarographic electrodes have often been used to validate new techniques aimed at assessing tumor hypoxia (18). However, this technique remains invasive and cannot be used to map tumor heterogeneity or to repeat measurements on the same site for a long time. Alternative methods are therefore required.

## Positron Emission Tomography

Hypoxia PET imaging is a non-invasive technique widely used in preclinical and clinical studies. This method requires the intravenous injection of a radiotracer (e.g., nitroimidazole) that will diffuse into cells and will be reduced intracellularly. This is reversible under normoxic conditions; but under hypoxia, the radiolabeled molecules will be trapped and will react with cellular macromolecules such as nucleic acids and proteins. The reduction requires the activity of reductases that are only present in viable hypoxic cells. As a consequence, the accumulation and detection of radiotracers will be enhanced in hypoxic regions, whereas the necrotic cells will not be visible to PET imaging. The quantification of the tracer uptake is generally expressed as the tumor-to-background (TBR) ratio at a given time after the tracer injection. 2-Nitroimidazoles have been developed as radiosensitizers (19). Because they have a nitro (NO_2_) group linked to the imidazole structure, they can undergo up to six electron reductions, eventually resulting in an amino group (NH_2_) (20). For PET imaging, these tracers are labeled with radioisotopes: fluorine-18 (^18^F) or carbon-11 (^11^C). The most important compounds designed to image hypoxia are described below.

### ^18^F-Fluoromisonidazole

*^18^F-FMISO* is a commonly used hypoxia tracer in preclinical and clinical studies. Due to its lipophilicity, this molecule easily crosses the cell membranes and is then trapped if intracellular hypoxia remains below a threshold of 10 mmHg. The cellular clearance of ^18^F-FMISO is quite low in normoxic tissues, thereby hampering the contrast between normoxic tissues and moderate hypoxic tumor tissues. As a result, a TBR ratio of 1.2 is usually used to delineate regions of hypoxia after a minimum delay of 2 h (20, 21). The best signal-to-noise ratio has been observed 4 h after tracer injection (22). In a study of Mortensen et al. (23), it was not possible to correlate ^18^F-FMISO with Eppendorf electrodes in the clinical setting in head and neck tumors (23).

In preclinical and clinical studies, the level of hypoxia highlighted by ^18^F-FMISO has been correlated with the response to therapy and outcome (24). Non-hypoxic volume estimated using ^18^F-FMISO uptake showed significantly better local control after single-dose irradiation than hypoxic tumors in FaDu hSCC xenografts (25). In a recent review on PET imaging, Fleming and colleagues listed all the applications of ^18^F-FMISO in clinical trials (21). This tracer has been successfully used to image hypoxia in gliomas, head and neck, and breast and renal tumors. However, the use of ^18^F-FMISO in sarcomas, pancreatic cancers, or rectal cancers was compromised because of the non-specific accumulation of ^18^F-FMISO in normoxic surrounding tissues or because of insufficient tracer uptake. Four head and neck tumor studies were able to correlate one ^18^F-FMISO-related tumor parameter (T:Bmax, SUVmax, or T:Mmax) with disease-free survival or locoregional failure (21), whereas one study was not able to establish any correlation (26). To date, different TBRmax thresholds for stratification have been reported; therefore, standardized methods still need to be determined in multicenter studies (27). Tumor mapping of hypoxia with ^18^F-FMISO could be useful for planning intensity modulated radiation therapy (IMRT) on patients with head and neck cancers, since when the hypoxic regions are well delineated, it is possible to boost the dose delivered to those areas. ^18^F-FMISO maps were used for this purpose on two patients in a study from Lee and colleagues in 2008. With the knowledge of hypoxic areas that they gained, these authors were able to escalate the dose to 84 Gy for 10 patients. Moreover, they raised the delivered irradiation doses up to 100 and 105 Gy for two patients in hypoxic areas (28). A single-center trial combining multimodal hypoxia imaging, including ^18^F-FMISO, and IMRT in patients with inoperable stage III non-small cell lung carcinoma (NSCLC) tumors was started in 2012 (29). Recent data also suggest that selective dose painting to hypoxic tumor subvolumes requires adaptation during treatment (30). ^18^F-FMISO has also been used to monitor reoxygenation of the tumors during the course of radiotherapy: in 10 patients, a decrease in the uptake of ^18^F-FMISO was observed in eight tumors after the delivery of 20 Gy (31). The reoxygenation process has also been observed in patients with glioblastoma treated by fractionated radiotherapy and concomitant temozolomide administration (32). The results showed a significant decrease in tumor hypoxia attributed to the radiotherapy effect. Finally, it has been suggested that the hypoxic areas of the tumors are correlated with neovascularization and with the tumor metabolism rate in glioblastoma multiform (33). This conclusion comes from a preliminary study involving 10 patients who underwent magnetic resonance imaging (MRI) to evaluate tumor perfusion after the injection of a gadolinium-based contrast agent and several PET imaging protocols: the first of these was ^18^F-FMISO as a reporter of hypoxia and the second was l-methyl-11C-methionine (^11^C-MET), an amino acid whose uptake reflects tumor activity and which is currently used for glioma detection and grading (34).

### ^18^F-Fluoroazomycin-Arabinofuranoside and ^18^F-Flortanidazole

^18^F-fluoroazomycin-arabinofuranoside (^18^F-FAZA) is a more hydrophilic nitroimidazole that displays faster clearance from blood and normal tissues than ^18^F-FMISO. As a result, imaging tumor hypoxia with this radiotracer improves the signal-to-noise ratio. In a preclinical study on rhabdomyosarcoma, a correlation between ^18^F-FAZA uptake and actual values of pO_2_ measured by EPR has been established, reflecting quantitative aspects of the method (35). Moreover, ^18^F-FAZA seems to be predictive of the response to radiotherapy: less hypoxic rhabdomyosarcoma tumors (defined by a lower uptake of ^18^F-FAZA) demonstrated better local tumor control 90 days after radiotherapy than more hypoxic tumors (36). Similarly, a significant correlation between ^18^F-FAZA T/B ratio and tumor growth delay was found in 9L glioma (37). With regard to clinical applications, ^18^F-FAZA imaging has been successfully performed in gliomas, lymphomas, lung, head and neck, and cervical and rectal tumors (21). The results of the DAHANCA 24 trial on head and neck cancers have proven that ^18^F-FAZA uptake is a good prognostic factor of tumor response to radiotherapeutic treatment (38). Finally, ^18^F-FAZA-PET images have been successfully exploited to delineate radiotherapy planning for head and neck squamous cell carcinoma, with 86 Gy being the dose to deliver in hypoxic areas. The treatment protocol included three phases and was based on ^18^F-FAZA-PET images acquired before irradiation and after the 7th and 17th fractions (39). ^18^F-flortanidazole (^18^F-HX4) is a hydrophilic nitroimidazole which quickly clears from normoxic tissues, allowing imaging 90 min after tracer administration; its low accumulation in the brain, heart, and gastrointestinal tract enables these body parts to be imaged (20). In a comparative study looking at several markers of hypoxia in an *in vivo* model (head and neck carcinoma cells SQ20b), ^18^F-FAZA, ^18^F-HX4, and ^18^F-FMISO uptakes were correlated with hypoxia, despite a relatively low accumulation of ^18^F-FAZA in muscles and tumors (40). In a second comparative study, ^18^F-HX4 and ^18^F-FAZA were found to be sensitive to an increase of hypoxia, induced by the breathing of a gas mixture containing 7% O_2_, when the tumor-to-blood ratio was used. However, when only the tumor-to-muscle was used, only ^18^F-FAZA revealed a significant decrease in tumor oxygenation (41).

### ^18^F-Fluoroerythronitroimidazole

^18^F-labeled fluoroerythronitroimidazole (FETNIM) was suggested as another marker of tumor hypoxia for use with PET in 1995 (42). Initial data suggested that ^18^F-FETNIM shows low peripheral metabolism, little defluorination, and possible metabolic trapping in hypoxic tumor tissue (43). ^18^F-FETNIM distribution has been positively correlated with ^18^F-FAZA in murine mammary tumors under normoxic and hyperoxic conditions (44). It has been tested in head and neck, lung, cervical, and esophageal clinical cancer studies, with significant correlations between patient outcome and either high fractional hypoxic volumes, F-FETNIM uptake, or baseline SUVmax (21). Cross-validation studies with other quantitative oxymetric markers are lacking. However, F-FETNIM has been compared to F-FAZA in the preclinical and clinical settings, with positive correlations (21, 44, 45).

### Copper (II) Diacetyl-Bis (N4-Methylthiosemicarbazone)

Copper (II) diacetyl-bis (N4-methylthiosemicarbazone) (Cu-ATSM) can be used as a radiotracer with ^60–64^Cu with variable half-times (46). This agent displays high lipophilicity and rapid clearance from normoxic tissues, thereby enabling imaging 30 min after its administration (47). In the absence of oxygen, the Cu(II) is irreversibly reduced to Cu(I) in viable mitochondria and therefore becomes trapped in hypoxic cells. In the study by Carlin and colleagues, the ^64^Cu-ATSM molecule displayed a better uptake in tumor than ^18^F-FMISO, ^18^F-FAZA, and ^18^F-HX4. However, its distribution within the tumor was not similar to the other tracers: the accumulation of ^64^Cu-ATSM was greater at the tumor periphery and the uptake was lower in the tumor center where perfusion was also reduced (40). The low accumulation of ^60^Cu-ATSM in the urinary tract makes it an ideal candidate for imaging pelvic organs. For example, the uptake of this radiotracer has been inversely correlated with the patient outcome (in terms of progression-free survival) for 38 patients with cervical cancer (48). Similar observations have been performed in head and neck, and rectal and lung tumors, as reviewed in (16) and in more recent studies in NSCLC and head and neck tumors (49, 50). Few preclinical studies have attempted to link Cu-ATSM uptake and outcome; one study of canine tumors was not able to establish any correlation (51). Planning of dose painting can also be achieved using Cu-ATSM, which detects the hypoxic regions in preclinical and clinical models (52–54). However, Cu-ATSM uptake does not only reflect hypoxia: in a study of six tumor cell lines, the maximum uptake was cell line dependent and was linked to the redox status of tumor cells. The retention of Cu was higher in cells with an abnormally reduced status (55). Moreover, the *in vitro* results demonstrated that hypoxia selectivity was optimal 30–60 min after the administration of Cu-ATSM, but this is a limiting factor for *in vivo* applications, since the distribution of the tracer during the first hour after its administration is limited by a reduced tumor blood flow. The latter imaging is suggested to be rather linked to the active transport of Cu alone that has been dissociated from the Cu-ATSM complex, although these observations are again cell line dependent (55). The fact that copper metabolism may also play a role in the uptake mechanism of ^64^Cu-ATSM was confirmed in a more recent publication showing similar contributions between ^64^Cu-ATSM and ^64^Cu-acetate (56). Further studies have demonstrated that Cu-ATSM uptake is not correlated with an increase in hypoxia (57, 58) or that Cu-ATSM uptake is not co-localized with hypoxia marked with immunohistochemistry (40, 59, 60). Only one recent study has concluded in favor of a positive correlation between tracer accumulation and hypoxia but not in both tumor models under study (61). It seems that Cu-ATSM is not a specific marker of tumor hypoxia, but it has been successfully correlated with the NADH and NADPH levels: Cu-ATSM uptake is rather observed in tumors with abnormally reduced status, which may or may not be linked to hypoxia (62, 63). Finally, another study by Vavere and Lewis investigated the link between Cu-ATSM uptake and the fatty acid synthesis pathway, which consumes NADPH, and correlated the level of fatty acid synthase with the Cu-ATSM uptake (64). Consequently, Cu-ATSM images cannot be interpreted in terms of oxygenation only and, although Cu-ATSM is predictive of radiotherapy outcome, it is unclear whether this is linked to tumor hypoxia.

## MRI Methods

### ^19^F-MRI

^19^F-MRI is a non-invasive method able to map tumor hypoxia quantitatively, after the injection of a perfluorocarbon emulsion. Calibration curves relating the longitudinal relaxation rate to pO_2_ can be acquired for a given temperature and a given perfluorocarbon (65) and used to map tumor oxygenation quantitatively. A major advantage of this calibration is the independent property of the absolute ^19^F signal intensity, linked to perfluorocarbon uptake. The fluorocarbon (PFC) relaxometry using echo planar imaging for dynamic oxygen mapping method developed by Mason and colleagues has been successfully used to monitor positive and negative changes in tumor oxygenation (65–68) as well as to map the heterogeneity of response to hyperoxic challenges within each tumor: it appears that well-oxygenated areas at baseline will display an increase in oxygenation earlier than hypoxic areas (65). Similarly, ^19^F-MRI mapping was able to monitor the effect of a radiosensitizer, *S*-nitrosocaptopril, which induces a significant increase in tumor pO_2_ from 20 to 60 min after its administration (69). Due to an acquisition time reduced to 1.5 min, Jordan and colleagues were able to monitor spontaneous oxygenation fluctuations in the range of 5–30 mmHg in transplantable mouse liver tumors and to identify hypoxia cycles in this model (70, 71). PFC can remain in the tumor and enables repeated measurements. The injection of perfluoro-15-Crown-Ether in Shionogi tumors, a murine mammary carcinoma which has acquired a dependence on androgens, has shown that ^19^F-MRI is able to distinguish three hormone-dependent oxygenation statuses (72). Tumor tissue heterogeneity can be assessed by the diffusion-based multispectral technique in order to distinguish tumor necrosis from viable tumor tissue and to detect subcutaneous adipose tissue. In a recent study, Shi and colleagues monitored the tumor response to hyperoxic and hypoxic challenges by considering the tumor as a whole or by considering each tissue type separately. The pO_2_ increased significantly when the authors considered the tumor as a whole, and this response was enhanced further when they focused on the viable tumor tissue (73). However, in a study comparing ^18^F-PET imaging and ^19^F-MRI, it was shown that fluorine mapping with MRI was less sensitive to small pO_2_ changes (from 3 to 5 mmHg) in some tumors (35). Moreover, before performing ^19^F-MRI, the toxicity of the chosen PFC needs to be taken into account since it has been observed, for example, that the early toxicities (thrombosis and tissue necrosis) observed with HFB could be avoided by using 15C5 (74). Despite this, approval is being awaited from the FDA for the investigation in a clinical trial of PFCs as a biomarker of tumor response to radiotherapy.

### Dynamic Contrast-Enhanced Magnetic Resonance Imaging (DCE-MRI)

Dynamic contrast-enhanced MRI is a method widely used in preclinical and clinical research to assess information on tumor hemodynamics. A bolus of gadolinium-based contrast agent is injected, and its distribution within the tissue of interest is analyzed through signal enhancement, thereby providing information on perfusion and permeability. This technique is regularly combined with oximetric methods such as PET imaging, blood oxygen level-dependent (BOLD)-MRI, or EPR oximetry to assess tumor hemodynamic parameters and their impact on therapy (75–77). Two parameters are regularly assessed by DCE-MRI, using the Tofts model: *k*^trans^, representing the volume transfer constant between blood plasma and extravascular extracellular space, and *v*_p_, defining the blood plasma volume per unit volume of tissue. Low-perfused tumor areas suffer from an insufficient supply of oxygen, thereby leading to hypoxia. It has therefore been suggested that DCE-MRI could be used as an indirect method to detect hypoxic areas in tumors. In a recent preclinical study by Øvrebø and colleagues, *k*^trans^ was found to be predictive of tumor response to radiotherapy: low *k*^trans^ was associated with an increased radioresistance in hypoxic melanoma xenografts, suggesting that DCE-MRI is a biomarker of tumor radioresistance in hypoxic tumors (78). Further studies on two cervical cancer xenografts have confirmed those results, indicating that the radiotherapy outcome can be correlated with *k*^trans^ values measured before the treatment taking each tumor model separately (79). Furthermore, skewness in the distribution of the *k*^trans^ parameter was also correlated with poorer patient outcome, highlighting the heterogeneity of perfusion within these tumors (80). Conversely, a study on fibrosarcoma was not able to show any correlation between DCE-related parameters (*k*^trans^, *v*_p_, *K*_ep_, % of perfused voxels) and the outcome of radiation therapy (81). With respect to validation of the technique with other oximetric methods, attempts have been made in preliminary clinical studies to assess correlations between the level of hypoxia and permeability, with mixed results, in head and neck cancer and gliomas using pimonidazole staining (an immunohistological staining aimed at detecting tumor hypoxia) and in cervical cancer using polarographic electrodes (82–84). Søvik and colleagues have also demonstrated that DCE-MRI could be used to monitor the changes in tumor oxygenation during the course of radiotherapy in order to adapt IMRT to changes in hypoxia distribution within the tumor after several doses of irradiation (85). The Brix model can also be used for the analysis of DCE-MRI images. Perfusion or permeability is then assessed by the parameter *A*_Brix_, also known to measure the extravascular extracellular space (86). In order to compare the Brix and Tofts models and their ability to predict the outcome of patients, Andersen and colleagues tested both models on patients with cervical cancers. They concluded that low values of *k*^trans^ and *A*_Brix_ can be associated with poor outcome (87). A recent study also demonstrated that low *A*_Brix_ could be correlated with an upregulation of genes involved in the response to hypoxia (88).

Nevertheless, numerous precautions have to be taken when interpreting DCE-MRI images in terms of oxygenation because perfusion is not the only feature influencing pO_2_. Moreover, despite the establishment of relations between *k*^trans^ and oxygen tensions or immunohistochemical measurements in some studies, estimates of perfusion remain indirect estimates of hypoxia and, in some circumstances, do not relate to hypoxic status (89). It is also important to mention that the contrast agent distribution can also be altered by perfusion and extracellular volume, leading to misestimation of oxygenation in necrotic areas (85).

### Blood Oxygen Level-Dependent Magnetic Resonance Imaging

Blood oxygen level-dependent MRI, or fMRI, uses endogenous contrast and is sensitive to the effective transversal relaxation rate of protons $\left(R_{2}^{*}=\text{1}/T_{2}^{*}\right)$. This measurement is sensitive to the ratio of oxyhemoglobin and deoxyhemoglobin, the latter being a paramagnetic agent that shortens $T_{2}^{*}$.

In preclinical studies, BOLD-MRI has proven its ability to monitor changes in oxygenation levels during hyperoxic challenges (90–92). Our group has compared the changes in BOLD signal following the administration of carbogen or isosorbide dinitrate and has observed that the magnitude of the changes is stronger during the carbogen challenge (affecting the hemoglobin saturation) than after the administration of the NO donor (93). The oxygenation concentration is not the only parameter that affects $R_{2}^{*}$: changes in tumor blood flow, blood volume, blood pH, or metabolic status can also influence the $R_{2}^{*}$ measurements (94, 95). Changes in $R_{2}^{*}$ should therefore be carefully considered when they are treated as an indicator of changes in tumor oxygenation. Moreover, no correlation has been established between $R_{2}^{*}$ measurements and absolute values of pO_2_ (96). In comparisons with pimonidazole staining, both correlation and inverse correlation have been observed in prostate and mammary tumors, respectively (97, 98). BOLD-MRI is therefore used to monitor tumor oxygenation changes rather than to map tumor hypoxia quantitatively. Spontaneous fluctuations have been successfully monitored by BOLD-MRI, which has led to the identification of several cycles of hypoxia, with periods ranging from 3 min to 1 h (99). Moreover, the tumor regions in which tumor oxygenation fluctuates have been related to areas with functional vasculature. The prognostic value of $R_{2}^{*}$ was investigated in a preclinical study. The authors subjected rats with GH3-prolactinomas and mice with RIF-1 fibrosarcomas to carbogen breathing before radiation therapy. The GH3 prolactinomas displayed large $R_{2}^{*}$ and a large response to the hyperoxic challenge ($\Delta R_{2}^{*}$), and this was predictive of a transient reduction in the tumor size after irradiation. However, the inhibition of tumor growth exhibited with the RIF-1 fibrosarcoma was smaller, and this was related to a low $R_{2}^{*}$ at baseline and to a poor response to the hyperoxic challenge (100). Recently, our group observed that $R_{2}^{*}$ was predictive of radiation therapy outcome in rat 9L-glioma tumors but not in rhabdomyosarcoma tumors (101). In a study by Kim and colleagues, the results from a small sample of cancer cervical patients suggested that the $R_{2}^{*}$ values are predictive of the radiotherapeutic response (102).

It is important to remember that the BOLD signal is related to the amount of deoxyhemoglobin and therefore linked to the blood pO_2_ and blood saturation of oxygen (SO_2_). There is therefore a real interest in quantifying the BOLD signal (103). A technique known as multiparametric quantitative BOLD has been recently developed to achieve a quantitative mapping of tissue oxygenation. The method is based on the acquisition of several images, with standard sequences, aimed at measuring the blood volume fraction, field inhomogeneities (by mapping B0), and the tissue *T*_2_ before and after the administration of a contrast agent. These three values are then integrated in a model describing the $T_{2}^{*}$ signal in order to calculate average oxygen saturation in each voxel (95). This method has been successfully applied to map tumor hypoxia in the brain in stroke or gliomas (104). This approach allows a quantitative measurement of the blood oxygen saturation and represents a major improvement in the use of BOLD imaging to map tumor hypoxia and to monitor tumor oxygenation changes.

### ^1^H Relaxation Imaging

Oxygen is a paramagnetic agent that shortens the longitudinal relaxation time (*T*_1_) of surrounding protons. Consequently, *T*_1_ mapping appears as a possibility for mapping tumor hypoxia. This method does not require any contrast agent and is widely available in medical imaging centers. Moreover, a correlation can be established between pO_2_ values and relaxation rates, as was done, for example, between relaxation rates and arterial blood oxygen pressure in a pig (105), revealing the quantitative aspect of such measurements. However, since *T*_1_ relaxation is also influenced by temperature, tissue of interest, blood flow, and basal blood oxygen saturation, a calibration between *T*_1_ values and pO_2_ cannot be established so easily. Nevertheless, assessing *T*_1_ measurements has been a useful method for monitoring changes in tissue oxygenation.

In a recent study by Muir and colleagues reporting the use of a hyperbaric chamber for rodents, *T*_1_ values measured in the brain were found to be significantly reduced (with an increase in related *R*_1_ values) when successive switches were made from normobaric air to hyperbaric air and then to hyperbaric oxygen (106). On the basis of a comparison between the changes in *T*_1_ values in liver, kidney, and muscle of healthy rats obtained during transitions from air to pure oxygen, to carbogen (10% CO_2_ and 90% O_2_), or to a mixture of ambient air with 10% CO_2_, it was concluded that these measurements were sensitive to oxygen dissolved in tissues when there was no concomitant change in blood flow (107). However, in the same study, the authors highlighted the lack of sensitivity of *T*_1_ measurements to a decrease in oxygenation during a switch from pure oxygen to carbogen or from air to a mixture of air and 10% CO_2_. The explanation may lie in the vasodilatation induced by CO_2_, which offsets the decrease in tissue oxygenation, resulting in unexpected positive *R*_1_ changes (107). However, this issue is controversial: in a preclinical study aimed at evaluating brain oxygenation during a hyperoxic challenge, the *R*_1_ (*R*_1_ = 1/*T*_1_) values were similarly increased in the cerebral cortex and in the pituitary gland during both carbogen and pure oxygen breathing, despite a decrease in brain perfusion induced by pure oxygen breathing (indeed, in the absence of CO_2_, vasoconstriction occurs) (108).

*T*_1_ measurements have been assessed together with $T_{2}^{*}$ for monitoring tumor oxygenation in murine prolactinoma models and prostate tumor xenografts undergoing a hyperoxic challenge. The changes in relaxation rates were found to be related to the basal oxygenation status: the most hypoxic tumors exhibited significantly reduced *R*_1_ values and significantly higher $R_{2}^{*}$ values $\left(R_{2}^{*}=\text{1}/T_{2}^{*}\right)$ (109).

*T*_1_ measurements have recently been used in conjunction with perfusion MRI to quantify the hypoxic fraction in multiple models with differing hypoxic and vascular phenotypes (110).

In a preliminary human study on healthy volunteers, this technique was successfully used to monitor the increase in oxygenation in normal myocardium, spleen, and arterial blood. However, there was no significant change observed in liver, skeletal muscle, or subcutaneous fat (111). These discrepancies in the results were attributed to a lack of sensitivity, to the uncontrolled motion of organs, and to differences in blood flow, blood volume, and regional oxygen consumption. Two years later, a study by Noseworthy and colleagues also failed to monitor the oxygenation in skeletal muscles subjected to a hyperoxic challenge involving pure oxygen with *T*_1_ measurement despite a significant change in *T*_2_ (112). Nevertheless, further work demonstrated the effect of oxygen in shortening the longitudinal relaxation time in healthy volunteers’ muscles, spleen, renal cortex, subcutaneous fat, placenta, and liver (113–116). Furthermore, in another study by O’Connor and colleagues, the authors observed significant differences of response induced by either pure oxygen or carbogen depending on the tissue of interest: carbogen induced a lower *T*_1_-shortening effect than pure oxygen in the spleen, whereas the opposite phenomenon was observed in the liver. Ten patients with abdominal tumors were then subjected to pure oxygen breathing. A significant increase in *R*_1_ values was observed in eight of these patients with ovarian, cervical, or gastrointestinal malignancies (117). The effect of hyperoxia has also been investigated in the brain using a dynamic *T*_1_-weighted sequence called tissue oxygen level dependent (TOLD) (118). This method allows reduced acquisition times and is more suited for a dynamic assessment of changes induced by a hyperoxic challenge. Haddock and colleagues (119) monitored the oxygenation of brain tissue during two protocols: the first was composed of a twice-repeated switch from ambient air to pure oxygen with two 2-min intervals, while the second began with a normoxic phase followed by a switch to pure oxygen breathing during 7 min followed by a final breathing of air. Due to the *T*_1_ effect, they could observe changes in signal intensity whose magnitude was related to the changes in brain oxygenation monitored with BOLD-MRI. Furthermore, TOLD measurements have been demonstrated to be sensitive to dynamic hyperoxic challenges (120) and seem to be predictive of the radiotherapy outcome (92). Prostate tumors that are well reoxygenated during pure oxygen breathing before radiotherapy display a significantly larger regrowth delay than low responders to hyperoxia (92). Combined BOLD and TOLD contrasts were also recently assessed in Dunning rat prostate tumors and were shown to be useful prognostic factors for predicting tumor response to hypofractionation (121). However, these results remain preliminary and need to be further investigated. The study by Burrell and colleagues highlights the complementary character of concomitant *T*_1_ and $T_{2}^{*}$ measurements: although the mean response was an increase in *R*_1_ values and a decrease in $R_{2}^{*}$ values in both models, they observed that the magnitude of *R*_1_ and $R_{2}^{*}$ changes was dependent on the basal oxygenation status. The explanation may be linked to hemoglobin saturation: in well-oxygenated tumors, the increase in oxygen supply will raise the amount of dissolved oxygen rather than increase the already well-saturated hemoglobin. This results in higher amplitude changes of positive Δ*R*_1_ rather than of negative $\Delta R_{2}^{*}$ (109). It is also important to remember that the $T_{2}^{*}$ measurement is influenced by the hemoglobin and deoxyhemoglobin ratio and is therefore sensitive to vascular oxygenation, whereas the *T*_1_ measurement is rather sensitive to oxygen dissolved in tissues. As there is a real interest in combining the measurement of *R*_1_ and $R_{2}^{*}$, Ding and colleagues have recently proposed a new sequence enabling simultaneous acquisition of $T_{2}^{*}$ and *T*_1_ measurement. With this new method, they monitored the dynamic changes in oxygenation of abdominal organs (spleen, medulla, and renal cortex) (118).

From the foregoing, we can conclude that there is real value in measuring the ^1^H relaxation time to obtain information on tissue oxygenation non-invasively. By comparing the sensitivity of ^1^H-MRI oximetry with ^19^F-MRI using perfluorocarbons, Tadamura and colleagues observed that: “*T1 shortening effect with oxygenation observed using ^19^F MR system with PFCs is much greater than that observed with ^1^H MRI because of large oxygen solubility of PFC compared with that of aqueous media. Therefore, the PFC method is more sensitive to tissue oxygenation state*.” (111). In all the techniques described above, the signal was mainly influenced by protons of the water molecules. As oxygen solubility is higher in lipids than in water, focusing on the relaxation of protons belonging to lipid molecules would improve the sensitivity of the techniques described above. Consequently, a new technique called mapping of oxygen by imaging lipids relaxation enhancement (MOBILE) aimed at selectively measuring the *T*_1_ of the lipid component has been proposed (122). This method has demonstrated its ability to distinguish the oxygenation levels in tumor tissue homogenates submitted to different oxygen concentrations. Moreover, the MOBILE technique can be used to map local pO_2_ in several tissues such as liver, muscles, brain (infarcted or not), and mammary tumors. Both positive and negative changes in tumor oxygenation can be monitored with the MOBILE technique. Its quantitative aspect has also been demonstrated on mammary tumor models presenting high lipid content (123). However, the method was not applicable to tumors with low lipid content. An alternative method based on the deconvolution of global *T*_1_ in fat and water components was recently developed. However, a quantitative aspect could not be demonstrated in rhabdomyosarcomas and glioma models, and lipids *T*_1_ and global *T*_1_ turned out to be less predictive of the outcome of radiation therapy than $R_{2}^{*}$ (101). Considering the clinical application of MOBILE, the method has demonstrated its ability to identify hypoxia in stroke areas (124). The method is currently being investigated in human gliomas, with a pilot study showing that global *R*_1_ and lipid *R*_1_ values are significantly lower in tumors than in the “normal appearing white matter” of patients or the healthy brains of volunteers and that lipid *R*_1_ measurements enable discrimination between tumor areas and peritumoral edema (125).

Using the exogenous source of contrast hexamethyldisiloxane (HMDSO), R. P. Mason’s group identified a source of oxygen-sensitive contrast, called “proton imaging of siloxanes to map tissue oxygenation levels,” which was validated using hyperoxic breathing challenge in Dunning prostate R3327 MAT-Lu tumor-implanted rats but was not assessed as a predictive marker of the outcome of radiation therapy (126).

## Electron Paramagnetic Resonance

Quantitative assessments of tumor partial pressure of oxygen can be obtained with EPR. This magnetic resonance technique is sensitive to paramagnetic species (molecules presenting unpaired electrons). Because of the insufficient amount of radical species in viable tissues, EPR oximetry requires the injection of a paramagnetic probe into the site of interest. Particulate probes can be injected in the tumor once and used for repeated measurements during several months, with a high sensitivity (changes lower than 0.2 mmHg can be detected with this method) (8). The interactions between the two unpaired electrons of oxygen and the paramagnetic probe will lead to a change in *T*_2_ that can be observed by a change in the linewidth of the EPR spectrum acquired. The measurements themselves are non-invasive and enable the real-time monitoring of oxygenation changes over several hours or more. However, these measurements are restricted to surface tissues: *in vivo* EPR is performed with “L-band” spectrometers, operating at 1 GHz or less, allowing the penetration of microwaves up to a 10-mm maximum depth into the tissue (127). Although EPR spectrometry provides no anatomical information on tumor hypoxia, it has been successfully used in several tumor models to monitor changes in oxygenation levels induced by an increase in oxygen delivery (128–136), or by an inhibition of tumor consumption (76, 81, 137–143), or both (69, 144–149). As a quantitative technique, EPR oximetry is also predictive of tumor response to radiotherapy and can also be applied to monitor tumor reoxygenation after the administration of a radiosensitizer in order to determine the best therapeutic window in which radiotherapy should be performed (81). In 2010, Khan et al. showed that carbogen-induced reoxygenation of F98 glioma, assessed using EPR oximetry, significantly increased the tumor growth delay after radiation therapy (150). Also, in a study on C6 glioma, a first irradiation was applied and the changes in tumor oxygenation were assessed by EPR oximetry. Some tumors grew up to 150% of their basal oxygenation level. After a second irradiation, the well-reoxygenated tumors had a significant tumor growth delay compared to tumors whose response to the first irradiation was less than 50% from the baseline. In the same study, a second tumor model (9L glioma) did not exhibit an increase in tumor oxygenation after the first irradiation and remained radioresistant (151). Finally, in a study assessing the effect of benzyl nicotinate, EPR oximetry provided dynamic information on the changes in tumor pO_2_, which could be used to identify responders and non-responders and schedule therapy during the experiments (152). For the moment, the clinical application of EPR spectroscopy is just starting and restricted to three centers owning prototypes of human EPR equipment (153).

Electron paramagnetic resonance imaging is more challenging. Because of the fast relaxation of paramagnetic species (a matter of nanoseconds), most of the EPR experiments use EPR in a “Continuous Wave” mode: unlike in MRI measurements, the sample is submitted to a constant electromagnetic radiation and the measurement is performed by sweeping the magnetic field in order to reach the resonance condition. This increases the acquisition time. However, EPRI has demonstrated its ability to image oxygenation levels quantitatively *in vitro* and to map tumor hypoxia in preclinical models using a triarylmethyl probe (154, 155). A few researchers have developed home-made “Pulsed” EPR systems that work in the same way as MRI scanners available nowadays.

This makes shorter acquisition times possible and allows the repeated mapping of tumor hypoxia during spontaneous fluctuations or during hyperoxic challenges (156). In order to correlate the pO_2_ maps with anatomical information and perfusion measurements, researchers have developed a coil enabling EPR and MR imaging (157). By imaging tumor hypoxia with pulsed EPRI, Matsumoto and colleagues were able to follow tumor reoxygenation after the administration of sunitinib (a multi-tyrosine kinase inhibitor) after 4 days of treatment. They combined this chemotherapy with irradiation and concluded that the tumors subjected to a combination of these two therapies had a longer growth delay than tumors in mice receiving one of the therapies separately (158). The EPR measurements are mainly based on the transversal relaxation rate of the spin probe, but they can also be influenced by the probe concentration. A new method was successfully developed to address this issue: Epel and colleagues recently published a paper in which they measured the spin probe longitudinal relaxation rate with pulsed EPRI and a triarylmethyl probe which improves pO_2_ images by virtually eliminating the sensitivity to triarylmethyl concentration (159).

Finally, progress has been made in improving the sensitivity of MRI by exploiting the paramagnetic resonance: the use of Overhauser-enhanced MRI (OMRI) can increase the sensitivity of ^1^H measurement. OMRI is based on a double resonance principle: a paramagnetic agent (an EPR sensor) is first hyperpolarized and then a transfer of electron polarization occurs toward the surrounding water’s protons. As a result, the image intensity is enhanced. For oximetric measurements, Oxo63 (a soluble paramagnetic sensor also used in EPRI) can be used and the signal enhancement can be interpreted in terms of oxygenation. The Overhauser enhancement corresponds to the amplitude of the enhanced signal; it depends on the linewidth of the paramagnetic agent, which in turn depends on the oxygen concentration (160). Consequently, OMRI is a quantitative method that is able to monitor tumor oxygenation: it enables the detection of tumor reoxygenation during a carbogen challenge, and the pO_2_ values assessed at baseline as well as during the carbogen breathing are in agreement with pO_2_ obtained with Eppendorf electrodes in the same tumors (161). More recently, Oxo63 has been proposed as a marker of both hypoxia and permeability: since the molecular mass of Oxo63 is three times higher than that of gadolinium complexes (usually used as perfusion markers), its blood-to-tissue transfer will reflect permeability rather than perfusion. By analyzing the dynamic enhancement of ^1^H-MRI after Oxo63 administration to squamous cell carcinoma tumor-bearing mice, the authors concomitantly assessed tumor perfusion (the image’s increased contrast being proportional to the contrast agent concentration) and oxygenation (the image’s enhancement being inversely correlated with the oxygen concentration) (162). Further developments need to be achieved before this technique can be implemented in a clinical setting. In addition, the current research involving OMRI is restricted to a small number of laboratories, since the equipment is not common. For the moment, the major limitation is the undesired heating of the sample due to the saturation pulse.

## Conclusion

Several techniques are available to estimate tumor hypoxia. The pO_2_ measurements assessed with polarographic electrodes have been correlated with treatment outcome in both preclinical and clinical studies. However, this invasive technique is unable to provide maps of tumor hypoxia to plan radiotherapy. PET imaging is the most widespread method used in preclinical and clinical studies. It is involved in the delineation of the targeted volumes for radiotherapy planning. However, this method requires the injection of a radiotracer, and the imaging can only be achieved after a delay to allow tracer accumulation in hypoxic areas. Pulsed EPR imaging is of great interest in assessing tumor hypoxia in preclinical models. However, the instrumentation for pulsed EPR in preclinical conditions is restricted to a few imaging laboratories and the lack of clinical EPR imagers as well as the injection of an EPR sensor limit its applications. MR methods such as $T_{2}^{*}$ measurements and *T*_1_ measurement are promising, since they use oxygen as an endogenous contrast agent and can be easily implemented on all MRI scanners. Nevertheless, further studies are needed to investigate whether the relaxation times can be established as biomarkers of hypoxia and, more importantly, as predictive markers of radiotherapy outcome.

## Author Contributions

FC contributed to the redaction of the main text and drew the figure; BG revised the manuscript; BJ contributed to the redaction, revision of the main text, and computed data for the table.

## Conflict of Interest Statement

The authors declare that the research was conducted in the absence of any commercial or financial relationships that could be construed as a potential conflict of interest.
